# Predictive factors for beneficial application of high-frequency electromagnetics for tumour vaporization and coagulation in neurosurgery

**DOI:** 10.1186/1477-7819-6-45

**Published:** 2008-04-29

**Authors:** Rainer Ritz, Stefan Heckl, Sam Safavi-Abbasi, Guenther C Feigl, Boris Krischek, Wolf Lüdemann, Javed M Mirzayan, Andrei Koerbel, Madjid Samii, Marcos Tatagiba, Alireza Gharabaghi

**Affiliations:** 1 Department of Neurosurgery, Eberhard Karls University, Tübingen, Germany; 2 Neurosurgery, International Neuroscience Institute, Hannover, Germany

## Abstract

**Objective:**

To identify preoperative and intraoperative factors and conditions that predicts the beneficial application of a high-frequency electromagnetic field (EMF) system for tumor vaporization and coagulation.

**Methods:**

One hundred three subsequent patients with brain tumors were microsurgically treated using the EMF system in addition to the standard neurosurgical instrumentarium. A multivariate analysis was performed regarding the usefulness (ineffective/useful/very helpful/essential) of the new technology for tumor vaporization and coagulation, with respect to tumor histology and location, tissue consistency and texture, patients' age and sex.

**Results:**

The EMF system could be used effectively during tumor surgery in 83 cases with an essential contribution to the overall success in 14 cases. In the advanced category of effectiveness (very helpful/essential), there was a significant difference between hard and soft tissue consistency (50 of 66 cases vs. 3 of 37 cases). The coagulation function worked well (very helpful/essential) for surface (73 of 103 cases) and spot (46 of 103 cases) coagulation when vessels with a diameter of less than one millimeter were involved. The light-weight bayonet hand piece and long malleable electrodes made the system especially suited for the resection of deep-seated lesions (34 of 52 cases) compared to superficial tumors (19 of 50 cases).

The EMF system was less effective than traditional electrosurgical devices in reducing soft glial tumors. Standard methods where also required for coagulation of larger vessels.

**Conclusion:**

It is possible to identify factors and conditions that predict a beneficial application of high-frequency electromagnetics for tumor vaporization and coagulation. This allows focusing the use of this technology on selective indications.

## Background

A wide range of electrosurgical devices has been developed during the last decades to facilitate tumor removal and/or hemostasis in surgery [1-6]. As most of these electrosurgical instruments are based on unique principles, they have to be carefully evaluated with respect to the possibility of added functionality and efficacy as a surgical adjunct [4,7-11].

Recently, the clinical applicability of a new commercially available, high-frequency electromagnetic field (EMF) system has been demonstrated [12], and specific handling techniques and electrode tip configurations could be defined for optimal use [13].

However, there is still the necessity for a systematic evaluation of benefits and shortcomings of this radiofrequency electrosurgical unit in order to define its place in the standard surgical armamentarium for tumor resection. Therefore, this study was performed to identify preoperative and intraoperative factors and conditions that predict the beneficial application of this system in tumor vaporization and coagulation during brain surgery.

## Methods

### Patient Population

One hundred three subsequent patients with intracranial tumours including 38 meningiomas, 23 neurinomas, 19 gliomas, 12 metastasis, 5 adenocarcinomas, 4 chordomas, and 2 adenomas were treated surgically with the aid of the EMF system.

### Description of the EMF system

The system to be evaluated (Orion-1™ EMF System, Ortho Development Corp., Salt Lake City, Utah, USA) is an electrosurgical device operating at radiofrequency (13.56 MHz) with a maximum electrical output of 16 watts. [3] Based on the principles of electromagnetics, the system generates localized heat when the tips of the electrodes interact with underlying living tissue. Its functions are defined as vaporization, cutting, and coagulation controlled by depressing one of two foot pedals labeled "vapo/cut" and "coagulation", respectively. The system offers two disposable handpiece types (bayonet and straight) with disposable electrodes featuring six different electrode tips (1 and 2 mm ball, 3 and 5 mm ring, needle, and blade). The ball and ring tips are malleable. The electrode shafts are supplied in a long (95–100 mm) and in a short (55–60 mm) version.

### Evaluation of the EMF system

All microsurgical approaches were performed without modification of the surgical strategy for the purpose of this study; the EMF System was used as an adjunct or, at times, as a substitute for other instruments.

In each case, the whole range of EMF functionality was evaluated during surgery by interviewing the surgeon immediately after each maneuver. Thereby, all options (vaporization, cutting, and coagulation) have been explored in each case in the course of the study. Moreover, the coagulation mode was evaluated separately for surface bleeding, small vessels, and large vessels.

Immediately after operation, the surgeon rated the overall performance of the system in terms of effectiveness (ineffective vs. effective) and graded – in cases of effective application – its contribution to the surgical procedure (useful, very helpful, or essential). Thereafter, we performed a multivariate analysis including tumor histology and location, tissue consistency and texture, patients' age and sex. Statistical evaluation included Student's t test with paired and unpaired comparisons and Fisher's exact test. P values less that 0.05 were considered significant.

## Results

**Figure 1:**
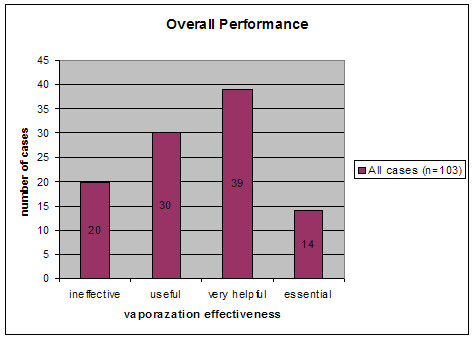
Overall EMF vaporization effectiveness (ineffective/useful/very helpful/essential) in 103 brain tumour cases.

The EMF system functioned properly in all 103 procedures and was not attributed to any complications. It could be used effectively during surgery in 83 of 103 cases, being useful, very helpful, and essential in 30, 39, and 14 cases, respectively (Fig. 1). Patients' age and sex did not correlate to any of the analyzed categories.

**Figure 2:**
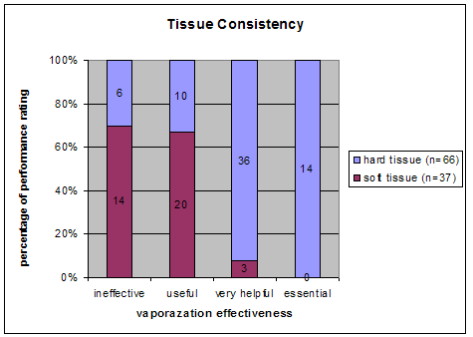
EMF vaporization effectiveness (ineffective/useful/very helpful/essential) comparing different tumour consistencies (hard/soft).

Sixty-six of all 103 lesions were classified as tumors with a hard consistency by the treating neurosurgeon. In 50 of these 66 cases, the contribution of the EMF system to the overall success was rated as very helpful or essential. In the group of tumors with a soft tissue consistency, there were only 3 of 37 cases in the same category of effectiveness (very helpful or essential) (Fig. 2). This difference between hard and soft tissue consistency in terms of effective EMF application was significant (p < 0.05).

**Figure 3:**
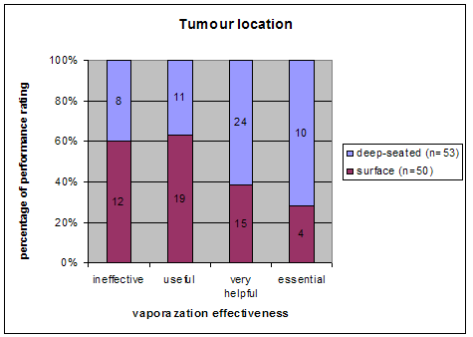
EMF vaporization effectiveness (ineffective/useful/very helpful/essential) comparing different tumour locations (deep-seated/surface).

Fifty-three of all 103 lesions were classified as deep-seated tumors. In 34 of these 53 cases, the contribution of the EMF system to the overall success was rated as very helpful or essential. In the group of tumors located at the surface, there were 19 of 50 cases in the same category of effectiveness (very helpful or essential) (Fig. 3). This difference between deep-seated and surface tumors in terms of effective EMF application was significant (p < 0.05).

**Figure 4:**
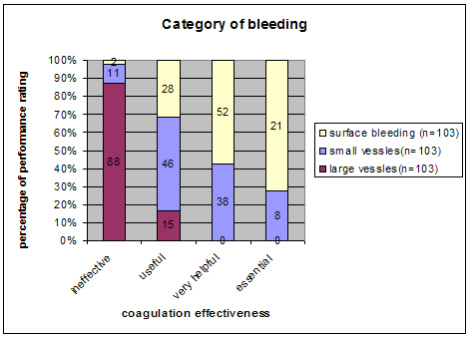
EMF coagulation effectiveness (ineffective/useful/very helpful/essential) comparing different bleeding categories (surface/small vessels/large vessels).

The coagulation function worked well (very helpful/essential) for surface (73 of 103 cases) and spot (46 of 103 cases) coagulation when vessels with a diameter of less than one millimeter were involved. In large vessels the EMF system was ineffective in 88 of 103 cases and was considered useful in only 15 of 103 cases (Fig. 4). This difference in terms of effectiveness regarding coagulation of larger vessels was significant (p < 0.05).

## Discussion

The effectiveness of the vaporizing function of the EMF system was directly affected by the location of the surgical field and the type of tissue involved.

The ergonomic design of the handpiece combined with a very light weight allowed for comfortable handling while operating on deep-seated lesions. The bayonet shape was completely compatible with the microscope and did not block the surgical view. When used in conjunction with the long malleable electrodes, the EMF system was found to be especially suited for long approaches to deep-seated lesions as both access and visibility were improved compared to surgery employing the shorter and more voluminous ultrasound aspirator. The ability to use a single-tipped probe on tumors compressing the brain stem through narrow approaches without adverse affects caused by heat or current undoubtedly adds to the standard surgical armamentarium.

Recent findings indicate that electromagnetic field and local inductive hyperthermia have an impact on tumor growth [14]. Though, it has to be emphasized that there is a difference between radiofrequency hyperthermia producing heating of a whole tumor, and an electromagnetic devise which is used for surgical tumor resection by creating pinpoint heat at the probe tip. This focused effect is induced in the present study by applying a sharp convergence of the current with maximum current density at the probe tip, resulting in minimal spread of heat, and the capability to vaporize tissue in a very focal way [5,12,15,16].

The EMF system performed best in harder tumors. With rubbery or fibrous tumors the electrodes did not have difficulty cutting through the tissue for dissection. However, the device was not effective for resection of highly calcified tumors. In future a preoperative computed tomography may help to avoid unnecessary application of the system in calcifies lesions.

Moreover, the EMF system was less effective than traditional electrosurgical devices in reducing soft glial tumors. In soft or cystic tumors, the system's electrodes would become enveloped in tumor and the thermal reaction would be stifled. Therefore, at our institutions the EMF systems are not any longer used for glial tumors.

When the ball tips where used together with the coagulation mode, surface coagulation was possible and proved most effective. The coagulation mode could also be used for cutting purpose with ring, needle and blade tip electrodes. When working in vascular tissue it was possible to use the coagulation mode for a cut with a surrounding peripheral zone of coagulation in a number of cases. Thus an incision could be made without the need to switch tools.

In large vessels the EMF system was not effective. In these cases, standard methods where required for coagulation. In highly vascularized tumors, the effectiveness of the EMF System was also greatly hampered. In these cases, bipolar coagulation had to be used predominantly.

The necessity to switch to an ultrasonic aspirator when resecting soft tumor tissue or to a bipolar forceps when coagulating larger vessels may present as a major drawback of the present EMF system. Therefore, at present electrosurgical units based on electromagnetic fields are not able to replace the standard devised, but may serve as a valuable tool in specific indications.

## Conclusion

This new electrosurgical device adds to the armamentarium for the resection of hard and fibrous tumors especially in small approaches to deep-seated lesions. The coagulation function worked better for surface and spot coagulation when vessels with a diameter of less than one millimeter were involved. A thorough understanding of the indications and principles of the new technique is required for its effective use. It has the potential to attain a place as a complementary tool with additional functionality among the standard surgical equipment.

## Competing interests

The authors declare that they have no competing interests.

## Authors' contributions

RR and AG conceived of the study, implemented the technology, and participated in its design and coordination and drafted the manuscript. MS and MT participated in the coordination of the study and helped to draft the manuscript. SH, SSA and GCF participated in the design of the study and performed the statistical analysis. BK, WL, MJM and AK carried out the surgical application of the EMF tool, participated in the tumour and vessel categorizations, as well as in the postoperative interviews. All authors read and approved the final manuscript.
